# Prevalence Trends and Associated Factors of the Double Burden of Malnutrition Among Rwandan Women of Childbearing Age: Evidence From Demographic Health Surveys, 2010–2020

**DOI:** 10.1002/puh2.70270

**Published:** 2026-05-27

**Authors:** John Mugisha, Japhet Ishimwe, Theophile Dushimirimana, Joseph Imanishimwe, Liang Zhou

**Affiliations:** ^1^ Rwanda Implementation Science Kigali Rwanda; ^2^ College of Medicine and Health Sciences, School of Public Health University of Rwanda Kigali Rwanda; ^3^ Institute of Molecular Immunology, School of Laboratory Medicine and Biotechnology Southern Medical University Guangzhou China

**Keywords:** double burden of malnutrition, Rwanda, women of reproductive age

## Abstract

**Background:**

Double burden of malnutrition (DBM) is a significant public health concern among women of reproductive age globally. Overweight or obesity is more prevalent than underweight, leading to an increased risk of noncommunicable diseases and childbirth complications, contributing to maternal and infant mortality. The study aimed to assess prevalence trends and factors associated with DBM among Rwandan women.

**Methods:**

The cross‐sectional study used data from the Rwanda Demographic and Health Survey (RDHS) conducted in 2010 and 2020. It involved 20,926 women who were not pregnant and aged 15–49, with newborns at least 2 months old at the time of the survey. The analysis adjusted for sampling weights. BMI was calculated according to World Health Organization (WHO) classifications, and the study involved sociodemographic variables and household characteristics. Data analysis used descriptive statistics, chi‐square tests, and multivariable binary logistic regression with 95% confidence intervals (CIs) at a significance level of *p* ≤ 0.05.

**Results:**

Underweight among women decreased from 6.75% to 5.57%. Overweight/Obesity rose from 17.81% to 27.42%; normal nutrition declined from 75.44% to 67.01%. Women aged 20–24 had lower odds of being underweight; those aged 44–49 had higher odds (aOR: 2.929; CI: 1.661–5.164; *p* < 0.0001). Never‐married (aOR: 1.803; CI: 1.230–2.644; *p* = 0.003) and widowed/divorced women (aOR: 1.836; CI: 1.183–2.848; *p* = 0.007) had higher odds. Adventists were less likely (aOR: 0.580). The poorer and poorest categories showed higher odds of underweight (poorest OR: 2.353; CI: 1.356–4.082; *p* = 0.002). Overweight/Obesity increased with age, marital status, religion, occupation, and urban residence. Richest group aOR: 3.572 (CI: 2.600–4.907; *p* < 0.001); TV aOR: 1.365 (CI: 1.086–1.716; *p* = 0.008); radio aOR: 1.177 (CI: 1.018–1.361; *p* = 0.027).

**Conclusion:**

DBM is rising among Rwandan women. Overweight or obesity, driven by socioeconomic factors, requires targeted interventions, awareness campaigns, empowerment, and further research to inform effective public health policies.

## Graphical abstract

From 2010 to 2020, the prevalence of overweight or obese Rwandan women substantially increased from 18% to 27%, whereas that of underweight women decreased slightly. Overweight/Obesity increased with age, marital status, religion, occupation, wealth, and urban residence and media access, whereas poverty, being unmarried, widowed/divorced were associated with underweight.

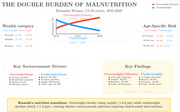

## 1 Background

The World Health Organization (WHO) defines the double burden of malnutrition (DBM) as the simultaneous occurrence or manifestation of both overnutrition and underweight among individuals at various stages of life, within the same household, and or within the same population [1, 2, 3].

Underweight occurs when an individual's BMI is below 18.5 kg/m^2^ [4]. Although obesity is defined as a BMI of 30.0 kg/m^2^ or higher, a BMI of 25.0–29.9 kg/m^2^ is considered overweight [5, 6, 7, 8, 9]. Overweight or obesity has nearly tripled in the past 30 years [10]. This condition has been linked to several noncommunicable diseases (NCDs), including cardiovascular disease, stroke, and type 2 diabetes mellitus [11, 12, 13].

According to the WHO, in 2020, 462 million adults suffered from underweight, 1.9 billion were classified as overweight/obese, with over 600 million in the obesity category, and 30% of women aged 15–49 were anemic worldwide [14, 15, 16]. Overweight or obesity has become more frequent in many developing countries over the past decade, whereas underweight has decreased due to demographic and nutritional transitions. The impact has been greater on women than on men [17, 18, 19, 20]. As a result, inadequate nutrition in pregnant women, mothers, and their children contributes to 11% of the worldwide disease burden [4], leaving them more prone to DBM than men [21, 22, 23].

DBM is considered a significant public health concern among women of reproductive age globally, with rising rates in developing countries, including Rwanda [17, 24, 25]. The DBM affects almost every country in the Pacific and Sub‐Saharan Africa, as well as regions of Southern Asia [19, 26, 27, 28]. DBM significantly strains governments, households, and individuals, who are all under considerable financial and physical strain [29].

Aged women with excessive nutritional intake are more likely to develop NCDs and other conditions, such as gestational diabetes, preeclampsia, bleeding, and the need for cesarean delivery, as well as maternal and infant mortality during childbirth, according to previous studies conducted [30, 31]. The global emergency of DBM has been linked to demographic changes, epidemiology, and nutrition [32].

Rwanda, located in central Africa, has an approximate prevalence of 33.2% for the DBM from 2010 to 2020 [33, 34, 35]. Rwanda has invested in resources and strategies to combat malnutrition. In 2008, Rwanda implemented performance‐based financing to improve Rwandans’ health [36]. Demographic change is causing populations with high birth and death rates, particularly among younger individuals, to shift toward older populations more susceptible to DBM [14, 37]. Wars, conflicts, hunger, and famines were also factors influencing DBM [38]. However, there is little understanding of the factors that are associated with DBM among women of childbearing age in Rwanda.

### 1.1 Objectives of the Research

The research aimed to assess prevalence trends and factors associated with DBM among Rwandan women of childbearing age (15–49 years).

## 2 Methods

### 2.1 Study Design

The research employed a cross‐sectional design.

### 2.2 Sampling Procedures

The study used data from the Rwanda Demographic and Health Survey (RDHS) and employed a two‐stage sampling design. The study obtained the primary sampling frame by collecting data from the Rwanda Housing Census conducted in 2002 for the 2010 survey and in 2012 for the 2015 and 2020 surveys [33, 34, 35]. In the first stage, enumeration areas (EAs) were selected with probability proportional to size from all rural and urban areas in all provinces of Rwanda, resulting in 492 villages as clusters in 2010 and 2015 and 500 villages in 2020 [33, 34, 35]. In the second phase, 26 households per cluster were systematically selected from updated household lists in each EA using an equal‐probability sampling method [33, 34, 35]. Women who met the eligibility criteria were invited to participate in the survey.

### 2.3 Inclusion and Exclusion Criteria

This study included all females aged 15–49 years who were not pregnant, had no birth within 2 months of the survey, had complete BMI data, consented to the DHS survey, and had no health conditions that would prevent participation.

### 2.4 Data Collection Procedures

Data collectors were trained healthcare technicians who used standardized offline interviews on tablets. Data collectors were assessed via in‐class activities, quizzes, and observations conducted during field practice. Later, anthropometric measurements, such as weight and height, were taken to calculate BMI, and this was done explicitly by healthcare providers.

### 2.5 Study Population and Sample Size

Women aged 15–49 were included, and the research used the women's recode (IR) datasets, including only women with BMI data who were nonpregnant and delivered more than 60 days before the survey. The study specifically targeted permanent residents to ensure representation and generalizability and used sampling weights to mitigate potential bias from unequal selection probabilities [39, 40]. In this research, we used a two‐stage stratified cluster design, incorporating sampling weights at each stage and for each cluster. The study used weighted sample sizes of 6976 in 2010, 6685 in 2015, and 7265 in 2020, totaling 20,926 respondents.

### 2.6 Study Variables

The DBM represents anthropometric measurements of both underweight (low BMI) and overweight or obese individuals. BMI for women aged 15–49 is calculated by dividing their weight in kilograms by their height in meters squared. WHO classifies BMI as underweight (<18.5) or normal (18.5–24.9). The classification for overweight is a BMI range of 25.0–29.9, whereas obesity is defined as a BMI of 30.0 or higher [2, 9, 41]. Independent variables were maternal age, education level, residence type, region, respondents who were currently working, occupation title, children born, number of household members, breastfeeding, marital status, religion, and wealth index calculated by DHS using principal component analysis, radio access, access to television, toilet facility type, water drinking source, and contraceptive use [42, 43, 44, 45, 46, 47, 48, 49].

### 2.7 Data Analysis

The data analysis involved utilizing the statistical software STATA/SE version 15.1 to process and analyze the data. To account for over‐ and undersampling in the study, the survey command was used to adjust the output to reflect the existing population samples accurately. The analysis included a detailed table presenting descriptive statistics and trends in nutritional status, along with crude odds ratio and multivariable binary logistic regression analyses. Additionally, the *χ* ^2^ test was used to assess the independence of the explanatory variables, and regression and variance‐factor indexes were used to assess multicollinearity [50, 51, 52]. The Hosmer–Lemeshow test was applied to determine how well our model fits [53, 54, 55]. Using the most recent dataset, multivariable binary logistic regression was used to test for significance between the predictor and explanatory variables (*p* < 0.05) and to estimate odds ratios and 95% confidence intervals (CIs) [56].

## 3 Results

**FIGURE 1:**
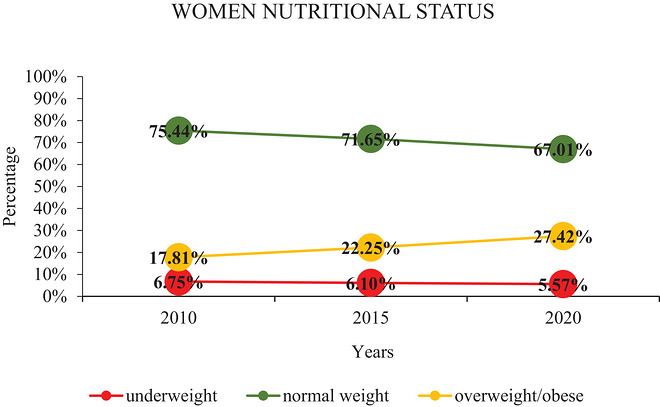
Overall prevalence trend of DBM in women of reproductive age in Rwanda.

Table 1 indicates the overall nutritional status of study respondents within the years 2010 (*N* = 6976), 2015 (*N* = 6685), and 2020 (*N* = 7265). The prevalence of underweight was 6.75% in 2010, 6.1% in 2015, and 5.57% in 2020, respectively. These data had a slight decrease of about 1.18% in underweight prevalence in the past 15 years, whereas overweight or obesity showed a significant increase from 17.81% in 2010 to 22.57% in 2020 (Table 1 and Figure 1). The data indicate an increase in the prevalence of overweight or obesity by approximately 10% over the past decade, highlighting a greater burden of this issue among Rwandan women of childbearing age, which requires comprehensive intervention to address this public health problem. The chi‐square and *p* value (158.9853, *p* < 0.001) indicated a statistically significant association between DBM and overall years.

**TABLE 1:** Overall nutritional status of women of reproductive age in Rwanda from 2010 to 2020.

Years	Underweight	Normal	Overweight or obesity			
	*N* (%)	*N* (%)	*N* (%)	Total	*χ* ^2^	*p* value
2010	471 (6.75)	5262 (75.44)	1243 (17.81)	**6976**	***χ* ^2^ = **158.985	**<0.001**
2015	408 (6.10)	4790 (71.65)	1488 (22.25)	**6685**		
2020	405 (5.57)	4869 (67.01)	1992 (27.42)	**7265**		
**Total**	1283 (6.13)	14,921 (71.30)	4722 (22.57)	**20,926**		

Bold texts indicate chi‐square and *p*‐values.

Figure 1 illustrates the prevalence trend of the DBM among women of childbearing age in Rwanda from 2010 to 2020.

### 3.1 Prevalence of DMB Distributed With Sociodemographic Characteristics

Table 2 summarizes the sociodemographic and prevalence distributions of nutritional status among women of reproductive age in this study. There was a significant association between age and nutritional status among the women (*p* < 0.001). The prevalence of underweight women aged 15–19 years declined from 12.35% in 2010 to 10.32% in 2020, whereas the prevalence of overweight or obesity increased among women aged 30–34 years and 45–49 years, from 20.67% and 17.56% to 32.76% and 27.87%, respectively. The educational level was significantly associated with DBM (*p* < 0.001). The women with the highest education had a lower percentage of underweight, with an increase in overweight or obesity, from 44.3% in 2010 to 53.55% in 2020. Women with no education experienced an increase in underweight status, rising from 5.7% to 7%.

**TABLE 2:** Sociodemographic characteristics and distribution of the nutritional status of women of reproductive age.

	RDHS 2010			RDHS 2015			RDHS 2020		
Characteristics	Underweight *n* (%)	Normal *n* (%)	Overweight or obesity *n* (%)	Underweight *n* (%)	Normal *n* (%)	Overweight or obesity *n* (%)	Underweight *n* (%)	Normal *n* (%)	Overweight or obesity *n* (%)
**Demographic factors**									
1. Maternal age								***χ* ^2^ = **608.33	***p* < 0.001**
15–19	191 (12.35)	1174 (76.03)	180 (11.63)	147 (10.56)	1045 (75.2)	198 (14.24)	167 (10.32)	1207 (74.49)	246 (15.19)
20–24	49 (3.523)	1063 (76.7)	274 (19.78)	41 (3.313)	931 (75.83)	256 (20.86)	39 (3.359)	818 (71.26)	291 (25.38)
25–29	63 (4.923)	976 (76.22)	242 (18.86)	48 (4.163)	836 (72.41)	271 (23.43)	38 (3.783)	678 (67	294 (25.38)
30–34	36 (4.072)	668 (75.26)	183 (20.67)	49 (4.767)	698 (68.21)	277 (27.02)	35 (3.152)	712 (64.08)	364 (32.76)
35–39	37 (5.153)	535 (74.06)	150 (20.79)	43 (5.357)	522 (65.75)	230 (28.89)	32 (3.189)	630 (62.69)	343 (34.12)
40–44	45 (7.229)	453 (73.41)	119 (19.36)	33 (5.361)	424 (69.22)	156 (25.42)	34 (4.63)	429 (57.84)	278 (37.53)
45–49	50 (9.298)	395 (73.14)	95 (17.56)	147 (10.56)	1045 (75.2)	198 (14.24)	60 (9.46)	396 (62.67)	176 (27.87)
2. Marital status								***χ* ^2^ = **417.87	***p* < 0.001**
Never in relation	252 (9.12)	2110 (76.44)	399 (14.44)	207 (8.146)	1929 (75.73)	411 (16.12)	230 (7.96)	2110 (72.89)	554 (19.15)
Married/Partner	168 (4.84)	2606 (74.95)	703 (20.21)	141 (4.107)	2358 (68.63)	937 (27.26)	124 (3.33)	2311 (62.38)	1271 (34.29)
Widowed/Divorced	50 (6.84)	546 (74.04)	141 (19.12)	59 (8.393)	503 (71.6)	141 (20.01)	51 (7.68)	447 (67.21)	**167 (25.11)**
3. Religion								***χ* ^2^ = **117.98	***p* < 0.001**
Catholic	207 (6.97)	2312 (77.87)	450 (15.16)	183 (6.931)	1945 (73.75)	510 (19.32)	181 (6.89)	1797 (68.54)	644 (24.57)
Protestant	198 (6.95)	2103 (73.71)	552 (19.34)	172 (5.64)	2166 (70.94)	715 (23.42)	175 (3.64)	2302 (66.67)	966 (28.05)
Adventist	49 (5.11)	705 (74.11)	198 (20.78)	41 (5.228)	547 (69.96)	194 (24.81)	33 (3.64)	608 (66.67)	271 (29.7)
Moslem	5 (6.450)	53 (68.62)	19 (24.93)	8 (6.062)	82 (59.59)	47 (34.35)	9 (5.98)	80 (53.81)	59 (40.21)
Others	6 (10.33)	43 (72.18)	11 (17.5)	1 (1.56)	32 (68.53)	14 (29.91)	4 (4.22)	48 (55.16)	36 (40.63)
No religion	5 (10.48)	36 (68.38)	11 (21.14)	2 (8.578)	14 (65.1)	6 (26.32)	4 (6.89)	34 (62.71)	17 (30.4)
**Socioeconomic factors**									
1. Education level								***χ* ^2^ = **330.64	***p* < 0.001**
No education	73 (6.85)	818 (76.99)	172 (16.17)	598 (7.02)	144 (74.73)	800 (18.06)	42 (5.77)	518 (71.75)	162 (22.48)
Primary	335 (7.01)	3653 (76.4)	793 (16.59)	254 (5.89)	3161 (73.21)	902 (20.9)	229 (5.44)	2876 (64.8)	1100 (26.16)
Secondary	53 (5.11)	746 (72.25)	234 (22.65)	80 (5.795)	947 (68.47)	356 (25.74)	122 (6.02)	1341 (66.26)	561 (27.72)
Higher	10 (9.86)	45 (45.72)	42 (44.43)	15 (8.371)	84 (45.67)	85 (45.96)	13 (3.98)	134 (42.47)	**169 (53.55)**
2. Occupation								***χ* ^2^ = **875.67	***p* < 0.001**
Not working	129 (11.11)	855 (73.38)	181 (15.51)	98 (10.04)	661 (67.54)	219 (22.41)	182 (9.47)	1295 (67.32)	447 (23.21)
Professional	6 (5.382)	61 (52.55)	49 (42.07)	11 (5.94)	90 (50.63)	78 (43.42)	7 (3.89)	79 (42.14)	101 (53.96)
Clerical	2 (7.48)	17 (63.06)	8 (29.46)	1 (4.493)	16 (49.54)	14 (45.97)	1 (1.35)	27 (49.21)	27 (49.44)
Sales	12 (3.47)	200 (57.28)	137 (39.25)	14 (2.518)	298 (55.49)	226 (42)	0	0	0
Agriculture	241 (5.37)	3538 (78.79)	712 (15.84)	249 (5.73)	3328 (76.66)	764 (17.6)	110 (4.42)	1737 (70.01)	634 (25.56)
HH and domestic	9 (5.638)	102 (67.37)	41 (26.99)	10 (4.38)	150 (66.42)	66 (29.21)	7 (3.081)	142 (65.16)	69 (31.76)
Services	1 (3.10)	17 (50.16)	16 (46.74)	5 (4.21)	72 (59.41)	44 (36.38)	19 (2.53)	384 (52.11)	334 (45.36)
Manual works	70 (11.11)	463 (73.3)	98 (15.59)	19 (7.18)	173 (65.18)	73 (27.64)	80 (4.79)	1204 (72.4)	**380 (22.82)**
Wealth index							***χ* ^2^ = **1097.85	***p* < 0.001**	
Poorest	117 (9.31)	987 (78.39)	155 (12.31)	121 (9.25)	1025 (78.45)	161 (12.3)	95 (7.44)	1001 (78.14)	185 (14.42)
Poorer	109 (7.78)	1132 (80.68)	162 (11.54)	82 (6.24)	1022 (77.71)	211 (16.05)	101 (7.17)	1065 (75.56)	244 (17.27)
Middle	83 (6.03)	1100 (79.4)	202 (14.58)	78 (6.25)	954 (76.07)	222 (17.68)	76.7 (5.55)	1008 (72.92)	297 (21.52)
Richer	91 (6.49)	1040 (74.45)	266 (19.07)	55 (4.40)	899 (71.63)	301 (23.98)	75 (4.97)	926 (61.64)	502 (33.39)
Richest	70 (4.58)	1004 (65.55)	458 (29.87)	71 (4.58)	890 (57.25)	593 (38.17)	57 (3.39)	869 (51.39)	765 (45.22)
3. Respondents working							***χ* ^2^ = **101.77	***p* < 0.001**	
Yes	285 (5.72)	3768 (75.61)	930 (18.67)	280 (5.38)	3759 (72.38)	1155 (22.24)	200 (4.16)	3228 (66.98)	1391 (28.86)
No	184 (9.28)	1489 (74.99)	312 (15.73)	128 (8.62)	1024 (69)	332 (22.37)	205 (8.37)	1640 (67.06)	601 (24.56)
**Geographical factors**									
1. Residence type							***χ* ^2^ = **556.33	***p* < 0.001**	
Rural	401 (6.79)	4560 (77.12)	951 (16.09)	340 (6.34)	4037 (75.32)	983 (18.33)	356 (6.1)	4103 (70.31)	1377 (23.59)
Urban	69 (6.502)	702 (66.08)	291 (27.42)	68 (5.09)	753 (56.8)	505 (38.1)	49 (3.43)	766 (53.55)	615 (43.02)
2. Region of residence								***χ* ^2^ = 548.17**	***p* < 0.001**
Kigali	48 (5.90)	508 (62.43)	258 (31.67)	44 (4.94)	532 (59.27)	322 (35.8)	38 (3.59)	559 (53.11)	455 (43.3)
South	159 (9.93)	1247 (77.87)	195 (12.19)	138 (8.61)	1199 (74.79)	266 (16.59)	132 (8.69)	1070 (70.29)	320 (21.02)
West	94 (5.53)	1321 (77.42)	291 (17.05)	66 (4.54)	1084 (74.94)	297 (22.24)	93 (5.78)	1139 (70.97)	373 (23.24)
North	52 (4.43)	930 (78.87)	197 (16.7)	46 (4.22)	801 (73.46)	243 (22.32)	48 (4.37)	756 (69.4)	286 (26.23)
East	117 (6.98)	1255 (75.01)	301 (18.01)	113 (6.89)	1173 (71.26)	360 (21.86)	95 (4.73)	1345 (67.34)	558 (27.93)
**Household/Environment factors**									
Children born							***χ* ^2^ = **289.39	***p* < 0.001**	
None	240 (9.04)	2006 (75.58)	408 (15.38)	193 (8.27)	1740 (74.6)	399 (17.12)	212 (8.19)	1885 (72.56)	500 (19.25)
1–3	126 (5.59)	1698 (75.45)	427 (18.95)	104 (4.10)	1820 (71.79)	611 (24.11)	102 (3.63)	1817 (64.72)	888 (31.64)
4–6	68 (4.88)	1047 (75.36)	275 (19.76)	84 (6.20)	900 (66.55)	368 (27.25)	71 (4.99)	875 (61.51)	477 (33.5)
7 and above	37 (5.44)	511 (75.01)	133 (19.55)	27 (5.76)	330 (70.9)	109 (23.34)	19 (4.43)	292 (66.58)	**127 (28.99)**
1. HH members							***χ* ^2^ = **61.6154	***p***: 0.4424	
7 and above	132 (6.43)	1552 (75.78)	364 (17.79)	134 (7.72)	1191 (68.49)	414 (23.8)	109 (6.29)	1148 (66.17)	478 (27.54)
4–6	240 (6.94)	2596 (75)	625 (18.06)	203 (5.79)	2522 (71.98)	779 (22.23)	208 (5.14)	2725 (67.32)	1114 (27.54)
1–3	99 (6.72)	1114 (76)	253 (17.28)	71 (4.89)	1077 (74.66)	295 (20.45)	88 (5.92)	996 (67.13)	400 (26.95)
1. Toilet type								***χ* ^2^ = **159.98	***p* < 0.001**
Unimproved	130 (7.98)	1274 (78.35)	222 (13.67)	146 (8.05)	1387 (76.47)	281 (15.48)	122.1 (7)	1266 (72.64)	354.7 (20.36)
Improved	330 (6.37)	3858 (74.37)	999 (19.26)	252 (5.311)	3313 (69.72)	1187 (24.97)	275 (5.09)	3529 (65.35)	1597 (29.56)
2. Drinking water source							***χ* ^2^ = **406.47	***p* < 0.001**	
Improved	172 (6.37)	1906 (70.68)	619 (22.95)	182 (6.11)	1946 (65.37)	849 (28.52)	171 (4.35)	2460 (62.5)	1305 (33.14)
Others	14 (6.13)	183 (77.9)	37 (15.98)	8 (7.4)	84 (75.78)	19 (16.82)	8 (6.75)	76 (60.85)	41 (32.4)
Non improved	284 (7.02)	3169 (78.49)	585 (14.49)	218 (6.05)	2757 (76.7)	620 (17.25)	225 (7.029)	2332 (72.79)	647 (20.18)
**Access to media factors**									
1. HH with radio							***χ* ^2^ = **223.81	***p* < 0.001**	
No	166 (7.45)	1756 (78.72)	308 (13.82)	202 (7.22)	2145 (76.57)	454 (16.22)	244 (6.096)	2920 (72.98)	837 (20.92)
Yes	294 (6.41)	3378 (73.67)	913 (19.92)	197 (5.23)	2560 (67.87)	1015 (26.9)	153 (4.875)	1875 (59.67)	**1114 (35.45)**
2. HH with TV								***χ* ^2^ = **772.86	***p* < 0.001**
No	435 (6.91)	4818 (76.63)	1035 (16.46)	362 (6.42)	4198 (74.52)	1074 (19.06)	359 (6.11)	4165 (70.88)	1352 (23.01)
Yes	26 (4.89)	313 (59.63)	186 (35.47)	38 (4.03)	504 (53.78)	396 (42.19)	38 (2.98)	630 (49.71)	599 (47.3)
**Behavioral/Maternal health factors**									
1. Breastfeeding							***χ* ^2^ = **55.48	***p* < 0.001**	
No	361 (7.44)	3557 (73.36)	931 (19.2)	306 (6.39)	3386 (70.64)	1101 (22.97)	331 (6.07)	3603 (66.17)	1512 (27.76)
Yes	110 (5.17)	1706 (80.17)	312 (14.66)	102 (5.37)	1404 (74.19)	387 (20.44)	74 (4.09)	1266 (69.52)	480 (26.39)
2. Contraceptive use							***χ* ^2^ = **167.82	***p* < 0.001**	
Do not use	387 (7.43)	3921 (75.22)	905 (17.35)	340 (7.042)	3512 (72.85)	969 (20.11)	311 (6.63)	3236 (68.87)	1151 (24.51)
Use a modern contraceptive	83 (4.73)	1341 (76.1)	338 (19.17)	68 (3.652)	1278 (68.55)	518 (27.8)	94 (3.65)	1633 (63.61)	841 (32.74)

Abbreviation: RDHS, Rwanda Demographic and Health Survey.

**TABLE 3:** Variance inflation factor table.

VIF	Underweight	Overweight or obesity
Minimum	1.01	1.01
Maximum	3.23	3.39
**Mean VIF**	**1.71**	**1.76**

Women who were married or cohabiting reported the highest overweight or obesity rates, increasing from 20.21% in 2010 to 34.29% in 2020. Divorced or widowed women and women who have never been in a relationship also experienced small gains. Overweight or obesity was associated with urban residency (*p* < 0.001), with prevalence increasing from 27.42% in 2010 to 43.02% in 2020, compared to 18.33% to 23.59% in rural areas. In Kigali, the prevalence of underweight children rose from 31.67% to 43.3% between 2010 and 2020; however, in other regions, it declined overall. Occupation was significantly associated with the underweight prevalence of women (*p* < 0.001), with women in agriculture having a high and equal prevalence of overweight and underweight. Overweight among clerical workers increased from 29.46% in 2010 to 49.44% in 2020.

The number of children also influenced women's nutritional status (*p* < 0.001), as depicted by a decrease in underweight and an escalation of overweight or obesity in women with more children. The wealth index and DBM were significantly correlated (*p* < 0.001), and the prevalence of overweight among wealthier women was markedly higher (29.87%–45.22%). Access to media (TV, radio) was significantly associated with overweight and obesity (*p* < 0.001). Access to TV was associated with a prevalence of 35.68% in 2010 and 47.3% in 2020. The rate of modern contraceptive use was significantly higher in 2020, 32.74% among users versus 24.51% in nonusers. Regular feeding habits in contraceptive users declined from 76.1% in 2010 to 63.61% in 2020. The prevalence of overweight or obesity increased in both lactation and non‐lactation periods.

For the test, whose analysis was to determine multicollinearity between overweight or obesity as an outcome variable and other independent variables. Those eliminated had high multicollinearity: respondents currently working, regions, drinking water source, and toilet type. The minimum VIF for underweight regression analysis was 1.01, and the maximum VIF was 3.23, whereas the minimum VIF for Overweight or obesity regression analysis was 1.01, and the maximum VIF was 3.39 (Table 3).

Table 4 presents the multivariable binary logistic regression results for underweight among Rwandan women in 2020. The highest odds were observed among adolescents (15–19 years), and the lowest among women aged 20–24 (aOR = 0.454) and the older ones (44–49 years) (aOR = 2.928). Single (aOR = 1.803) and widowed/divorced (aOR = 1.836) individuals were at a higher risk of being malnourished. Adventist women were less likely to be underweight (aOR: 0.580) than Catholics. The likelihood of being underweight did not vary by unemployment among women; however, women in agriculture (aOR: 0.551), service (OR: 0.429), and manual jobs (aOR: 0.511) were less likely to be underweight.

**TABLE 4:** Multivariable binary logistic regression of factors associated with underweight among women of reproductive age in Rwanda in 2020.

Underweight	Odds ratio	Std. err.	*t*	*p* value	[95% conf. interval]
**Characteristics**					
*Demographic factors*					
1. Age of women					
15–19	Ref				
20–24	0.453	0.082	−4.380	<0.001	0.318–0.647
25–29	0.706	0.162	−1.520	0.129	0.450–1.108
30–34	0.611	0.166	−1.820	0.07	0.359–1.041
35–39	0.794	0.207	−0.890	0.376	0.476–1.325
40–44	1.396	0.387	1.200	0.23	0.809–2.407
45–49	2.929	0.845	3.720	<0.001	1.661–5.164
2. Marital status					
Married/Partner					
Never in relation	1.803	0.351	3.030	0.003	1.230–2.644
Widowed/Divorced	1.836	0.410	2.720	0.007	1.183–2.848
3. Religion					
Catholic	Ref				
Protestant	0.809	0.102	−1.680	0.093	0.631–1.036
Adventist	0.580	0.109	−2.900	0.004	0.401–0.839
Moslem	1.266	0.494	0.610	0.545	0.588–2.726
Others	0.603	0.459	−0.670	0.506	0.135–2.688
No religion	1.381	0.712	0.630	0.532	0.501–3.802
*Socioeconomic factors*					
1. Women's working status					
Yes	Ref				
No	1.105	0.272	0.400	0.686	0.681–1.792
2. Occupation					
Not working	Ref				
Professional/technical/managerial	1.111	0.548	0.210	0.831	0.421–2.930
Clerical	0.337	0.356	−1.030	0.304	0.042–2.683
Agriculture	0.551	0.152	−2.160	0.031	0.320–0.947
HH and domestic services	0.500	0.246	−1.410	0.159	0.190–1.314
Service providers	0.429	0.144	−2.520	0.012	0.222–0.829
Manual works	0.511	0.138	−2.490	0.013	0.301–0.867
3. Wealth index					
Richest	Ref				
Richer	1.366	0.359	1.190	0.236	0.815–2.289
Middle	1.673	0.468	1.840	0.066	0.966–2.900
Poorer	2.044	0.569	2.570	0.011	1.183–3.532
Poorest	2.353	0.660	3.050	0.002	1.356–4.082
*Geographical factors*					
1. Residence type					
Urban	Ref				
Rural	1.251	0.268	1.040	0.298	0.820–1.906
2. Region					
Kigali	Ref				
South	1.219	0.327	0.740	0.459	0.720–2.064
West	0.936	0.257	−0.240	0.809	0.546–1.604
North	0.648	0.190	−1.480	0.139	0.365–1.151
East	0.717	0.198	−1.200	0.229	0.417–1.234
*Household/Environmental factors*					
1. Children born					
None	Ref				
1–3	0.604	0.146	−2.080	0.038	0.376–0.972
4–6	0.523	0.174	−1.950	0.052	0.273–1.004
7 and above	0.346	0.130	−2.830	0.005	0.166–0.723
*Access to media*					
1. HH with TV					
Yes	Ref				
No	1.398	0.371	1.260	0.208	0.829–2.356
*Behavioral/Maternal health factors*					
1. Breastfeeding					
Yes					
No	0.747	0.138	−1.570	0.116	0.519–1.075
2. Contraceptive use					
Use modern contraceptives					
Don't use them	0.941	0.185	−0.31	0.758	0.640–1.385

The odds ratios for the lower wealth quantiles were three or more times those of the highest wealth quantiles, with the poorest having an aOR of 2.352. Women with one to three children were less likely to be underweight (aOR: 0.604), and those with seven or more children had lower odds. Media exposure and behavioral factors, such as what the child was fed (breast milk vs. other), were not consistently associated with underweight. In addition, the Hosmer–Lemeshow test assessed goodness of fit; the Hosmer–Lemeshow test result for the underweight child had a *p* value of 0.1513. We do not reject the null hypothesis, indicating that the model fits adequately.

Table 5 presents the multivariable binary logistic regression results for overweight or obesity among Rwandan women in 2020, with adolescents (15–19 years) as the referent. Women aged 20–24 years were 1.355 times more likely to be overweight or obese (95% CI: 1.047–1.753; *p* = 0.021), and the odds increased with age: 1.381 (*p* = 0.036), 1.461 (*p* = 0.017), 1.643 (*p* = 0.003), and 1.915 (p24:9) (*p* < 0.001). The odds of obesity were higher among urban women (aOR = 1.297; *p* = 0.004) and those with access to media as well: Women owning a TV (aOR = 1.365; *p* = 0.008) and those owning a radio (aOR = 1.177; *p* = 0.027) were more likely to be obese. There were also no significant differences in behavioral factors, including breastfeeding and contraceptive use. The Hosmer–Lemeshow test (*p* = 0.2585) validated the model's goodness of fit; the results for overweight or obesity indicated a *p* value of 0.2585. We do not reject the null hypothesis, indicating that the model fits adequately.

**TABLE 5:** Multivariable binary logistic regression of factors associated with overweight or obesity among women of reproductive age in Rwanda in 2020.

Overweight or obesity	Odds ratio	Std. err.	*t*	*p* value	[95% Conf. interval]
**Demographic factors**					
1. Age of women					
15–19	Ref				
20–24	1.355	0.178	2.310	0.021	1.047–1.753
25–29	1.382	0.212	2.110	0.036	1.022–1.868
30–34	1.461	0.232	2.390	0.017	1.070–1.995
35–39	1.643	0.276	2.960	0.003	1.182–2.285
40–44	1.915	0.347	3.590	<0.001	1.342–2.733
45–49	1.305	0.239	1.450	0.147	0.910–1.872
2. Marital status					
Never in relation	Ref				
Married/Partner	1.642	0.194	4.190	<0.001	1.301–2.071
Widowed/Divorced	1.289	0.198	1.650	0.100	0.952–1.743
3. Religion					
Catholic	Ref				
Protestant	1.237	0.089	2.940	0.003	1.073–1.425
Adventist	1.255	0.131	2.170	0.030	1.022–1.541
Moslem	1.485	0.323	1.820	0.070	0.969–2.278
Others	1.731	0.496	1.920	0.056	0.986–3.040
No religion	1.793	0.571	1.830	0.067	0.959–3.352
**Socioeconomic factors**					
1. Education level					
None	Ref				
Primary	1.233	0.150	1.710	0.087	0.970–1.567
Secondary	1.163	0.170	1.040	0.301	0.873–1.550
Higher	1.390	0.323	1.410	0.158	0.880–2.195
2. Occupation					
Not working	Ref				
Professional/technical/managerial	1.464	0.294	1.900	0.058	0.987–2.171
Clerical	1.376	0.451	0.970	0.331	0.722–2.621
Agriculture	1.085	0.106	0.840	0.404	0.896–1.314
HH and domestic services	1.053	0.168	0.320	0.746	0.770–1.440
Service providers	1.644	0.204	4.000	<0.001	1.288–2.099
Manual works	1.154	0.118	1.400	0.162	0.944–1.412
3. Wealth index					
Poorest	Ref				
Richest	3.572	0.577	7.870	<0.001	2.600–4.907
Richer	2.858	0.348	8.640	<0.001	2.251–3.630
Middle	1.602	0.191	3.950	<0.001	1.267–2.026
Poorer	1.299	0.164	2.080	0.038	1.014–1.663
**Geographical factors**					
Residence type					
Rural	Ref				
Urban	1.297	0.116	2.920	0.004	1.089–1.546
**Household/Environmental factors**					
1. Children born					
None	Ref				
1–3	1.263	0.164	1.800	0.072	0.979–1.630
4–6	1.331	0.203	1.880	0.061	0.987–1.796
7 and above	1.196	0.243	0.880	0.380	0.802–1.784
**Access to media**					
1. HH with TV					
No	Ref				
Yes	1.365	0.159	2.680	0.008	1.086–1.716
2. Radio					
No	Ref				
Yes	1.177	0.087	2.210	0.027	1.018–1.361
**Behavioral/Maternal health factors**					
1. Contraceptive use					
Don't use them	Ref				
Use modern contraceptives	1.063	0.083	0.790	0.430	0.912–1.239

## 4 Discussion

DBM is a global public health issue that is particularly prevalent in LMICs, such as Rwanda, where there has been a notable increase in its occurrence. This study aimed to assess trends in DBM prevalence and factors associated with DBM among women of reproductive age using data from the RDHS from 2010 to 2020. Rwanda, like other countries in Central Africa, has had a DBM prevalence of 33.1% for the past 15 years [33, 34, 35]. Like other SSA countries such as Ghana, the Democratic Republic of the Congo, Kenya, Nigeria, and Mozambique, it had a DBM, with 15–49‐year‐old women at 40.8%, 26.5%, 38%, 34%, and 23%, respectively [26].

This study's findings indicated a decrease in underweight, dropping from 6.75% in 2010 to 5.57% in 2020. However, overweight or obesity increased significantly, from 17.81% to 27.42%. This rise in overweight or obesity is of public health concern, as it is linked to a higher risk of NCDs, putting Rwanda at a higher double burden of disease [5, 18, 57, 58, 59].

Several factors were identified as linked to DBM. This study revealed factors associated with underweight, including age, marital status, socioeconomic status, religion, having children, and occupation. Additionally, factors like age, residence type, marital status, religion, occupation type, wealth index, and access to media tools like radio and television were associated with overweight or obesity.

The relationship between age and malnutrition is a significant public health concern, especially in LMICs, where older women are affected by both underweight and overweight or obesity. As people age, their bodies undergo changes that can affect their metabolism and nutritional status. However, several controversies exist in the existing literature. For instance, previous studies showed that aged women were more likely to be overweight or obese [60, 61, 62, 63, 64]. Amugsi et al. also reported that older age was significantly and positively associated with underweight [26, 57, 65]. Conversely, a study by Ikoona et al. indicated that women in the range of 25–35 years had high odds of being underweight; also, all ages were less likely to be overweight or obese [26]. Unlike previous studies with conflicting results, our research highlights the DBM among older women, emphasizing the importance of age‐appropriate nutritional interventions and policies to address both underweight and overweight or obesity risks in older populations.

Marital status has been increasingly identified as a significant factor of DBM, with contradicting findings across various contexts. A study by Song et al. revealed that married women had high odds of being overweight or obese, which is consistent with our findings [60, 63]. Supporting this trend, Ikoona et al. reported that married women had lower odds of being underweight [26, 57]. Additionally, research demonstrated that women from SSA who no longer lived with partners had lower odds of being underweight [62]. In contrast, women in relationships in 52 LMICs had no DBM [61].

Our analysis revealed that women who had never engaged in a relationship, as well as those who were widowed or had undergone divorce, demonstrated markedly high odds of being underweight. Conversely, married women or those cohabitating with partners had significantly higher odds of being overweight or obese. This could be because marriage may provide enhanced food security and stability within the household, thereby potentially influencing overweight or obesity risk. However, the circumstances of widowhood or divorce may lead to food insecurity and an elevated risk of underweight.

Religion has been identified as associated with DBM, but existing findings present controversial results regarding its role in this condition. Religious beliefs can definitely shape what and how people eat, how they take care of their health, and their overall way of living.

Consistent with our findings, previous studies have reported varying associations between religion and DBM. A study in Rwanda reported that Protestant women had higher odds of being overweight or obese, suggesting that some religious groups may have lifestyle or eating habits that contribute to being overweight or obese [66]. Conversely, a study conducted in SSA countries showed that religion was not a significant factor in either underweight, overweight, or obesity [62]. Additionally, religious beliefs and taboos may lead to underweight, primarily in communities where dietary constraints and restrictions limit access to a variety of meals [67]. Religious practices can influence overall health due to their principles, faith, and beliefs [67]. Our findings underscore the need to integrate religious and cultural contexts into public health strategies to address DBM through faith‐based programs.

Occupational status has been widely associated with DBM. Women who work in agriculture have direct access to food sources, which may help reduce the risk of underweight. Women who engage in manual labor and service provision also had reduced odds of being underweight, as they benefit from stable incomes and eventually have access to food. Opposing our findings, previous studies demonstrated that women with formal jobs had higher odds of overweight or obesity risk [30, 65]. Similar to our results, women in service provision positions had high chances of being overweight or obese [60]. This might be due to factors such as long hours spent in sedentary positions, limited access to healthy meals at work, and increased reliance on processed foods [68]. Therefore, it is essential that workplace initiatives promote physical activity and encourage healthier eating habits, especially for those in service fields.

Socioeconomic differences significantly influence nutrition and health outcomes. Women with lower incomes often suffer from food insecurity as a result of financial hardship. On the other hand, individuals with higher incomes face the opposite and tend to consume more calorie‐dense and processed foods, which may lead to overweight or obesity.

Our study found that women in the lowest wealth category had higher odds of being underweight, whereas those in the wealthiest group were often overweight or obese. As in previous studies, we found that wealth index was associated with DBM, especially among overweight or obese individuals [19, 57, 61]. Additionally, women in the richest and middle categories were highly associated with overweight or obesity [57, 63, 64, 66]. However, the study from Sierra Leone showed that wealthier women had lower odds of being overweight or obese [26]. Moreover, previous studies have shown that overweight or obesity is equally distributed across developed and developing countries [69, 70]. In Rwanda, cultural attitudes also play a significant role in how people view nutrition. For instance, being overweight or obese is often linked to wealth and prosperity, which can affect how individuals approach their diets.

Previous research yielded inconsistent results on the relationship between childbearing and maternal nutritional status. It was revealed that SSA women with children had lower odds of being underweight [62]. Our findings indicated that women with one to three children and those with seven or more had lower odds of being underweight. In contrast, there was no significant association between parity and the risk of overweight or obesity. This shows that in Rwanda, postpartum nutritional care and cultural feeding customs may act as protective factors against malnutrition. These findings highlight the importance of maternal nutrition interventions that balance the risks of overweight or obesity and underweight while taking into account sociocultural variables.

The media have influenced shifting eating habits, contributing to the rising prevalence of overweight or obesity in LMICs. Television and radio have an essential influence on food choices, with exposure to food and beverage advertisements, including those for high‐calorie, ultra‐processed foods, leading to changes in eating habits. Previous research on this link has yielded varied findings.

In line with our findings, previous research showed that women who accessed TV had higher odds of overweight or obesity risk [26, 57, 61]. These results highlight the possible influence of media exposure, as advertisements for unhealthy foods and drinks on television and radio might encourage consumption patterns that increase the risk of overweight or obesity [71]. Therefore, public health interventions should focus on media content controls and on using these platforms to promote nutrition and public awareness programs to combat overweight or obesity.

### 4.1 Strengths and Limitations of the Study

The research was reliable and valid due to the methodology used and the ethical considerations employed across all surveys. The datasets had missing BMI data, reducing the sample size and limiting the availability of essential variables, including physical activity, data composition, and granular dietary data. This study design has limitations in establishing causality. It is recommended that future research incorporate longitudinal data or interventional trials to strengthen causal inference.

## 5 Conclusions

The government of Rwanda has made significant investments in combating malnutrition, but DBM remains a public health problem in the country despite these efforts. Overweight or obesity is the most prevalent form of malnutrition. This study found that socioeconomic factors, household characteristics, and age were significantly associated with DBM among Rwandan women of childbearing age. Rwanda, in recent years, has focused on combating underweight in all age groups; it is now recommended that equal attention be given to addressing overweight or obesity. Strategies, such as bimonthly car‐free days, should target mostly specific groups, and raising awareness through health education and improved nutrition services can help in this regard. Furthermore, conducting additional epidemiological studies on DBM among women, focusing on all predictor variables, can help combat DBM by informing policymakers.

## Author Contributions

*Conceptualization:* John Mugisha and Liang Zhou. *Methodology:* John Mugisha and Liang Zhou. *Data preparation and formal analysis:* John Mugisha. *Writing of original draft:* John Mugisha, Japhet Ishimwe, Theophile Dushimirimana, and Joseph Imanishimwe. *Writing – review and editing:* John Mugisha. *Supervision and validation:* Liang Zhou.

## Funding

The authors have nothing to report.

## Ethics Statement

This study was observational, and secondary data were obtained from the Demographic and Health Survey. Permission to download the dataset was obtained from the Demographic Health Survey Program, with an authorization letter coded “AuthLetter_204797.” Therefore, this study did not require ethical approval or participant consent because the DHS program provided no personal identifiers or links.

## Consent

The authors have nothing to report.

## Conflicts of Interest

The authors declare no conflicts of interest.

## Data Availability

The data that support the findings of this study are available from the Demographic and Health Surveys (DHS) Program. Data are available from https://dhsprogram.com/data/Using‐DataSets‐for‐Analysis.cfm upon request and with the permission of the DHS program, subject to ethical and privacy restrictions.
